# Long-term administration of Tolvaptan to patients with decompensated cirrhosis

**DOI:** 10.7150/ijms.41454

**Published:** 2020-03-15

**Authors:** Kengo Kanayama, Tetsuhiro Chiba, Kazufumi Kobayashi, Keisuke Koroki, Susumu Maruta, Hiroaki Kanzaki, Yuko Kusakabe, Tomoko Saito, Soichiro Kiyono, Masato Nakamura, Sadahisa Ogasawara, Eiichiro Suzuki, Yoshihiko Ooka, Shingo Nakamoto, Shin Yasui, Tatsuo Kanda, Hitoshi Maruyama, Jun Kato, Naoya Kato

**Affiliations:** 1 Department of Gastroenterology, Graduate School of Medicine, Chiba University, 1-8-1 Inohana, Chuo-ku, Chiba 260-8670, Japan.; 2 Department of Gastroenterology and Hepatology, Nihon University School of Medicine, 30-1 Oyaguchi-Kamicho, Itabashi-ku, Tokyo 173-8610, Japan.; 3 Department of Gastroenterology, Juntendo University School of Medicine, 2-1-1 Hongo, Bunkyo-ku, Tokyo 113-8421, Japan.

**Keywords:** Tolvaptan, Cirrhosis, Ascites, HCC

## Abstract

**Aim**: Tolvaptan, an oral vasopressin-2 antagonist, sometimes improves hepatic edema including ascites in patients with decompensated cirrhosis. In this study, we examined the effectiveness and survival advantage in patients with the long-term administration of tolvaptan.

**Methods**: A total of 115 patients with refractory ascites who were treated with tolvaptan were retrospectively analyzed based on their clinical records. Patients with a decrease in body weight of ≥1.5 kg from the baseline on day 7 were determined as responders. Re-exacerbation was defined as a return to the baseline BW, dose escalation of conventional diuretics, or abdominal drainage.

**Results**: Of the 115 patients, 84 were included in this analysis. Response to tolvaptan treatment was observed in 55 out of the 84 patients (65.5%), with a mean weight reduction of 2.52 kg. Multivariate analyses demonstrated that body mass index (≥24) and urinary specific gravity (≥1.018) were significant predictors of the response to tolvaptan. However, cumulative re-exacerbation rates in responders at 6 and 12 months were 42.4 and 60.1%, respectively. Child-Pugh (classification C), HCC complication, and serum sodium levels (≥133 mEq/L) were determined as independent prognostic factors impacting overall survival (OS). Although there were no significant differences in OS between tolvaptan responders and non-responders, the responders without re-exacerbation within 3 months showed significantly longer OS than those with re-exacerbation within 3 months.

**Conclusion**: A persistent therapeutic response, but not early response to tolvaptan, was associated with favorable survival of decompensated cirrhotic patients.

## Introduction

Cirrhosis is an advanced stage of fibrosis of the liver caused by years of chronic injury mainly attributable to chronic infection of hepatitis B virus (HBV) and hepatitis C virus (HCV), alcohol abuse, and *non-alcoholic* steatohepatitis (NASH) 1. Although there are few symptoms in patients with *early-stage cirrhosis*, decompensated cirrhosis can cause many complications such as gastroesophageal varices, hepatic encephalopathy, and water retention 2. Among them, water retention including massive ascites is attributable to impaired activity of daily life 3. The primary care usually consists of treatment with conventional diuretics, such as loop diuretics and anti-aldosterone drugs in addition to salt restriction. However, it has been reported that dose escalation of conventional diuretics is associated with a poor prognosis because of hyponatremia and/or gradually progressing renal dysfunction 4. Although special treatment such as ascites puncture and drainage, cell-free concentrated ascites reinfusion therapy (CART), and peritoneovenous shunt placement is often performed in patients with refractory ascites, their effects are limited and temporary in most cases 5.

Cirrhosis has been reported to be commonly associated with high levels of renin-angiotensin, catecholamine, and vasopressin 6. Vasopressin is an antidiuretic hormone that increases the reabsorption of water by increasing water permeability through V2 receptors 7. Tolvaptan, a vasopressin selective V2-receptor antagonist, inhibits arginine vasopressin from binding to V2 receptors, and induces the excretion of electrolyte-free water without changing the total level of electrolyte excretion 8. Although previous studies revealed both the effectiveness and safety of tolvaptan administration over a comparatively short duration, further analyses involving long-term observation are extremely important to optimize the utility of this medical agent 9-11.

In this study, we assessed the impact of the long-term administration of tolvaptan. In addition, we investigated the relationship between re-exacerbation during treatment and the prognosis.

## Methods

### Patients and study design

**Figure 1:**
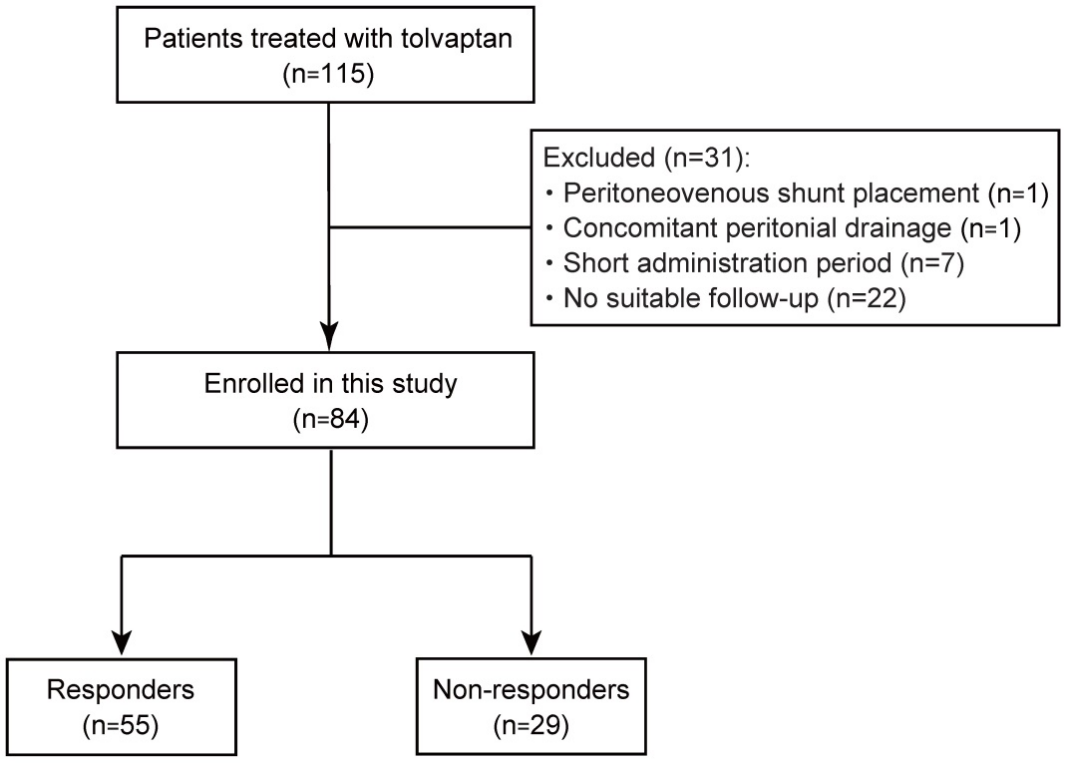
Study flow of patient enrollment.

A total of 115 patients with refractory ascites due to cirrhosis who were treated with tolvaptan, from January 2013 to September 2017, were enrolled in this study. The diagnosis of cirrhosis was based on the following criteria: (i) histological findings of biopsy specimens; (ii) imaging tests and endoscopic findings; and (iii) thrombocytopenia accompanied by findings suggestive of cirrhosis, such as splenomegaly 12. They had shown an insufficient response to conventional diuretics, furosemide and/or spironolactone. Thirty-one patients, including 1 patient with peritoneovenous shunt placement, 1 with intermittent abdominal drainage, 7 with cessation of tolvaptan, and 22 who had not undergone suitable follow-up, were excluded (Fig. 1). Ultimately, 84 patients were recruited for this study and their medical records were retrospectively analyzed. The study was conducted in accordance with the principles of the Declaration of Helsinski and approved by the Research Ethics Committees of the Graduate School of Medicine, Chiba University (approval number: 2656). All patients provided written informed consent for inclusion in the study.

Patients who had weight reduction of ≥1.5 kg in 7 days after the administration of tolvaptan were defined as responders according to previous studies 9-11. A return to the baseline body weight, dose escalation of conventional diuretics, or abdominal drainage was determined as re-exacerbation. Additionally, re-exacerbation within 3 months after tolvaptan administration was defined as early re-exacerbation.

### Statistical analysis

Statistical analyses were performed using JMP® pro 13 (SAS Institute Inc., Cary, NC, USA). Data are presented as medians and ranges. Statistical differences between two groups were evaluated by Wilcoxon rank sum test for continuous variables and chi-square test for categorical variables. Both re-exacerbation and overall survival (OS) were calculated using the Kaplan-Meier method. The significance of differences in OS between the two groups was evaluated by the log-rank test. P-values of <0.05 were considered significant.

## Results

### Patient characteristics

Of the 115 patients treated with tolvaptan for refractory ascites, 84 patients were considered eligible for this analysis (Table 1). They included 55 men and 29 women whose average age was 72.5 years (range, 29-93 years). Chronic liver damage was due to HBV (n=6), HCV (n=38), alcohol (n=12), and others (n=29). One patient showed coinfection with HBV and HCV. According to the Child-Pugh classification, they were classified as class A (n=2), class B (n=44), and class C (n=38). Forty-five patients (53.6%) were complicated by hepatocellular carcinoma (HCC). Three patients were accompanied by portal vein thrombosis and 6 with portal vein tumor thrombosis. The median daily dosages of furosemide and spironolactone before the initiation of tolvaptan were 20 mg (range, 0-160 mg) and 25 mg (range, 0-100 mg), respectively.

### Treatment with tolvaptan and response

The initial dose of tolvaptan was 3.75 mg in 37 patients (44.0%) and 7.5 mg in 47 patients (56.0%). The median administration period was 2.3 months (range: 0.3-60.0 months). A total of 79 patients (94.0%) were treated with tolvaptan for over 2 weeks. Although the effect was comparable between patients with tolvaptan of 3.75 mg and 7.5mg, the dose of tolvaptan in 29 out of the 37 patients who started with 3.75 mg of tolvaptan was subsequently increased. Among them, 10 patients discontinued the treatment because of an insufficient effect (n=3), a deteriorated renal function (n=3), hypernatremia (n=2), hepatic dysfunction (n=1), or ascites disappearance (n=1). After 7 days of tolvaptan treatment, 55 out of the 84 patients (65.5%) showed weight reduction of ≥1.5 kg with median weight reduction of 2.52 kg, indicating that they were responders to tolvaptan. Although the response rate of Child-Pugh C patients (71.1%) was modestly better than those in Child- Pugh A or B patients (60.9%), there was no significant difference. Considering that median serum urea nitrogen levels in Child-Pugh A or B and Child-Pugh C patients were 16 and 21mg/dL, respectively (p<0.01), this might be one of the causes of favorable response rate in Child-Pugh C patients. Concordant with this finding, 51 (68.0%) and 26 (56.5%) responders reported an improvement in the feeling of abdominal distension and lower leg edema, respectively.

### Markers predicting the effect of tolvaptan

To investigate the clinical factors affecting the response to tolvaptan, the patients were classified into responders (n=55) and non-responders (n=29). Clinical variables of each group were compared (Table 2). A positive response to tolvaptan was significantly correlated with a lower urea nitrogen level (*p*=0.03) and higher urinary specific gravity before administration (*p*=0.01). Subsequently, we examined the predictive markers of clinical variables. The variables with *p*<0.20 on univariate analysis (body mass index (BMI), serum levels of sodium, creatinine, urea nitrogen, albumin, and total bilirubin, and urinary specific gravity) were subjected to multivariate analysis. Multivariate Cox's regression analysis revealed that BMI (≥24) and urinary specific gravity (≥1.018) showed a significant correlation with the response to tolvaptan (Table 3). These results indicate that severe fluid retention and preserved renal function are required for a satisfactory tolvaptan response.

### Re-exacerbation after tolvaptan treatment

**Figure 2:**
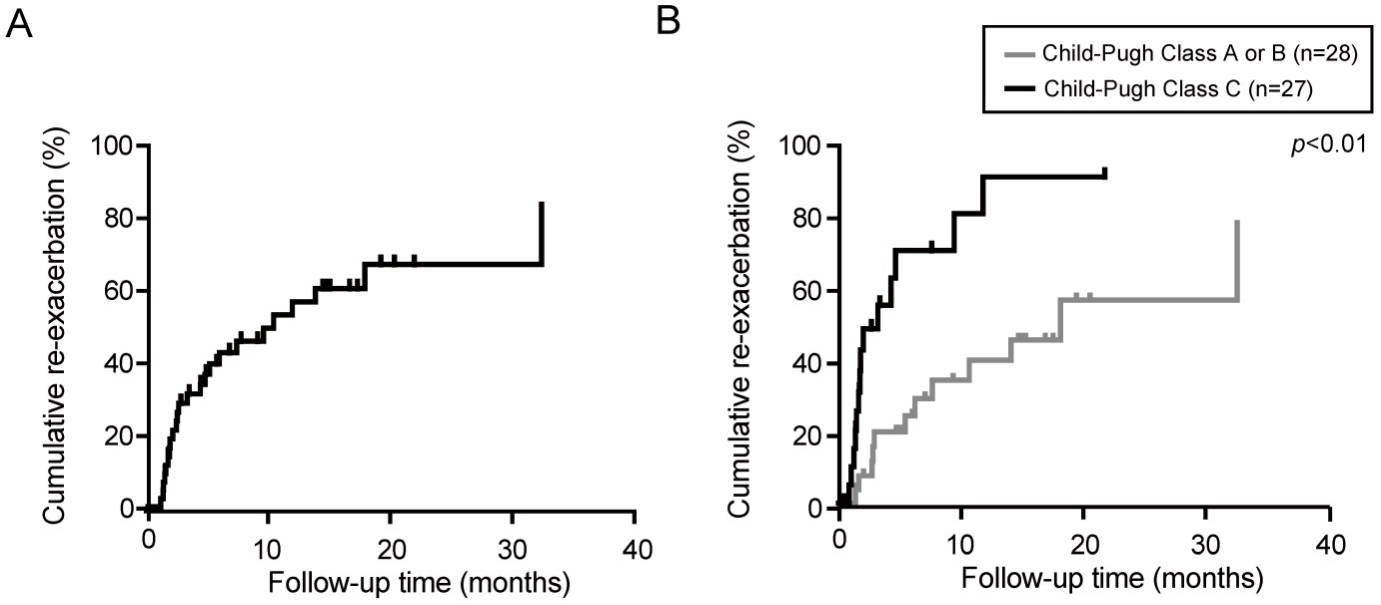
Re-exacerbation rate in responders to tolvaptan was estimated by Kaplan-Meier method. **(A)** Cumulative re-exacerbation rate in responders. **(B)** Cumulative re-exacerbation rate in Child-Pugh A or B patients and Child-Pugh C patients.

We then examined the persistence of the diuretic effect of tolvaptan in responders (n=55). The median observation period was 3.3 months (range, 0.3-40.5 months). The re-exacerbation rates were 42.4 and 60.1% at 6 and 12 months after the administration of tolvaptan, respectively (Fig. 2A). Eventually, 27 out of 55 responders demonstrated re-exacerbation. Among them, 15 patients had started with 3.75 mg of tolvaptan. The dose of tolvaptan in 13 out of 15 patients was escalated to 7.5mg before the re-exacerbation. Of importance, the re-exacerbation rates in Child-Pugh C patients were significantly higher than those in Child-Pugh A or B patients (*p*<0.01, Fig. 2B). The re-exacerbation within 3 months after tolvaptan administration (early re-exacerbation, n=14) was significantly correlated with a lower sodium level (*p*=0.04) and lower platelet count (*p*=0.04).

### Prognosis in view of tolvaptan response

**Figure 3:**
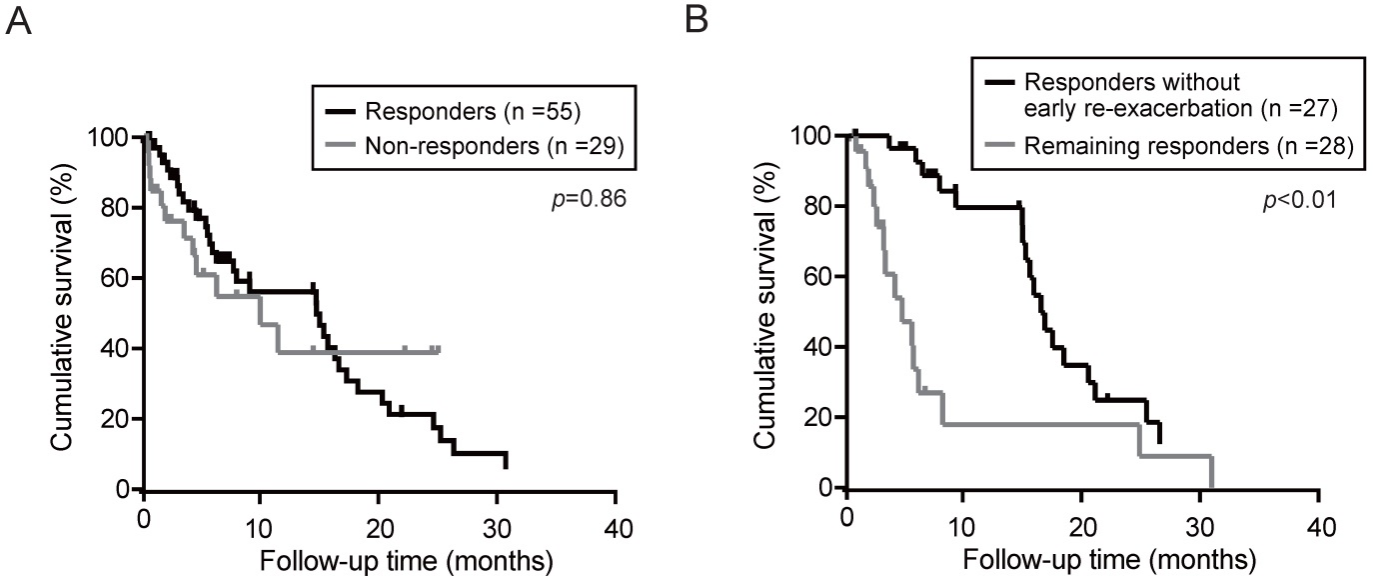
Overall survival (OS) in patients treated with tolvaptan was calculated by Kaplan-Meier method. (A) OS in responders and non-responders. **(B)** OS in responders without re-exacerbation within 3 months (early re-exacerbation) and the remaining responders.

Next, the overall survival in responders and non-responders was determined using the Kaplan-Meier method. Although the median OS in the responders and non-responders was 14.9 and 9.9 months, respectively, no significant differences was observed between the responders and non-responders (Fig. 3A). Even if limited in patients without HCC, there was no significant differences in OS between the responders (n=28) and non-responders (n=11) (*p*=0.30). To elucidate the prognostic relevance of clinical variables for OS, we performed univariate and multivariate analyses (Table 4). Multivariate analysis revealed that Child-Pugh (classification C), HCC complication, and serum sodium (≥133 mEq/L) showed a significant correlation with survival. Subsequently, we investigated the impact of re-exacerbation on OS in the tolvaptan responders. OS in the responders without re-exacerbation within 3 months after tolvaptan administration (early re-exacerbation) was significantly longer than those in remainders (Fig. 3B). Multivariate analyses demonstrated that not only HCC complication (*p*=0.02) but also early re-exacerbation (*p*<0.01) was identified as independent predictors for OS in responders (Table 5).

It has been reported that a decrease in conventional diuretic dosages was independent prognostic factor of survival in patients treated with tolvaptan for refractory ascites 13. We then examined OS in patients with dose-reduction of conventional diuretics (n=16) or without (n=39). Although the Kaplan-Meier analyses showed that OS of the patients with dose-reduction of conventional diuretics tended to be favorable compared with the patients without dose-reduction, there was no statistical significance (median OS; 16.2 vs 7.6 months, *p*=0.07).

## Discussion

Tolvaptan antagonizes a V2 vasopressin receptor and exerts a diuretic effect through the inhibition of water reabsorption in the renal collecting tubes 14. This agent is used for fluid retention refractory to conventional diuretics in patients with cirrhosis and congestive heart failure 15,16. As increased cyclic adenosine monophosphate production caused by V2 vasopressin receptor activation results in the promotion of autosomal dominant polycystic kidney disease (ADPKD), tolvaptan has also been utilized for the treatment of ADPKD 17. Tolvaptan, unlike furosemide and spironolactone, exhibits a diuretic effect without an increase in electrolyte excretion, although the agent often causes dehydration and hypernatremia 18. In this analysis, 2 patients (1.8%) developed hypernatremia and immediately discontinued the treatment.

At first, we examined the efficacy of tolvaptan. As 55 patients (65.5%) exhibited weight loss (≥1.5 Kg) within 7 days after tolvaptan administration, they were defined as responders according to the criteria proposed elsewhere. We then examined differences in characteristics between responders and non-responders. The responders showed a significantly higher BMI and urinary gravity than non-responders. Particularly, there were no significant differences in the comorbidity of HCC between responders and non-responders. Among the patients accompanied by HCC, the response rate to tolvaptan in The Barcelona Clinic Liver Cancer (BCLC) D stage patients were modestly lower than in BCLC A-C stage patients (data not shown). It is important to maintain the quality of life of patients with advanced HCC receiving best supportive care. Although tolvaptan may be useful as a tool for palliative care in advanced HCC patients complicated by decompensated cirrhosis. Considering that palliative medicine is concurrently performed during cancer treatment 19, tolvaptan should be administered before the progression to the BCLC-D stage.

We then conducted univariate and multivariate analyses of clinical variables affecting response to tolvaptan by multivariate Cox's regression analysis. BMI (≥24) and urinary specific gravity (≥1.018) showed a significant correlation with response to tolvaptan. Because most cases analyzed in this study were complicated by ascites due to decompensated cirrhosis, BMI indicated the levels of the body fluid retention, but not the degrees of obesity. It has been reported that an increase in vasopressin release was closely associated with the body water retention in cirrhotic patients and rats with experimental cirrhosis 20,21. Considering that tolvaptan is an active vasopressin V2-receptor antagonist, this agent might be effective in patients with high BMI rather than in patients with low BMI. Further analyses would be necessary to elucidate the relationship between serum vasopressin levels and effect of tolvaptan. It has been demonstrated that urinary specific gravity correlates well with urinary osmolality 22. We also previously reported that urinary specific gravity is associated with the response to tolvaptan 23. Similarly, Atsukawa et al. demonstrated that the blood urea nitrogen (BUN) level and urinary sodium excretion are closely associated with the response to tolvaptan 24. Given that urinary specific gravity reflects the renal concentrating ability, a maintained renal function is required to realize the diuretic effect of tolvaptan.

For a long time, diuretics such as spironolactone and furosemide were used for the treatment of ascites in patients with cirrhosis 25. However, the administration of furosemide and/or spironolactone causes an increase in serum BUN and creatinine levels and a decrease in the sodium level 26. Of note, it has been reported that cirrhotic patients with low serum sodium sometimes showed an unfavorable survival compared with patients with high serum sodium 27. Both the American Association for Study of Liver Diseases (AASLD) and European Association for the Study of the Liver (EASL) clinical practice guidelines recommended high-dose diuretic treatment (400 mg/day of spironolactone and 160 mg/day of furosemide as maximal doses) against refractory ascites 28. In contrast, the clinical practice guideline of the Japanese Society of Gastroenterology (JSGE) recommended additional tolvaptan (3.75 mg/day) administration for refractory ascites unaffected by 100 mg/day of spironolactone and 50 mg/day of furosemide 29. Together, clinical trials involving a large number of patients are needed to determine the timing of tolvaptan initiation.

Next, we focused on the time-course of responders. Of importance, approximately 50% of patients showed re-exacerbation within 12 months. In particular, Child-Pugh C patients showed a significantly higher re-exacerbation rate than Child-Pugh A or B patients. Although most patients continued tolvaptan treatment, they were treated with dose-escalated furosemide and/or spironolactone. Some of them concurrently received abdominal paracentesis. Taken together, it may be necessary to explore the therapeutic approach for ascites refractory to tolvaptan.

It is well-known that uncontrollable ascites may contribute to poor prognosis 30. However, tolvaptan improved the prognosis in some patients with decompensated cirrhosis 31,32. We subsequently calculated the OS in view of the response to tolvaptan. Unexpectedly, there were no significant differences between responders and non-responders. In fact, multivariate Cox's regression analysis revealed that Child-Pugh classification C, HCC complication, and lower serum sodium, but not the response to tolvaptan, were significantly correlated with the survival. Of importance, our analyses demonstrated that responders without re-exacerbation at least within 3 months showed longer OS than responders with re-exacerbation. Taken together, the persistence of a diuretic effect in patients treated with tolvaptan might contribute to a favorable prognosis. Further studies are necessary to confirm the effect of tolvaptan in a larger number of patients with respect to the survival benefit.

In summary, tolvaptan exerted an effect in more than half of cirrhotic patients within the first week after administration. The response to tolvaptan was closely associated with a good renal function. However, re-exacerbation, especially in Child-Pugh C patients, was frequently observed on long-term observation. The absence of early re-exacerbation, but not an early tolvaptan response, was associated with a favorable prognosis.

## Figures and Tables

**Table 1:** Baseline characteristics of the patients

Characteristics	Value (n=84)
Age (years)*	72.5 (29-93)
Sex (male/female)	54/29
BMI (kg/m^2^)*	24.4 (15.8-36.8)
Etiology HBV/HCV/alcohol/others	6 (7.1%)/ 38 (45.2%)/ 12 (14.3%)/ 29 (34.5%)
Child-Pugh Classification A/B/C	2 (2.4%)/ 44 (52.4%)/ 38 (45.2%)
FIB-4 index*	8.29 (2.26-26.0)
Complication of HCC	45 (53.6%)
Biochemistry	
Sodium (mEq/L)*	136 (120-145)
Potassium (mEq/L)*	4.1 (3.0-5.3)
Urea nitrogen (mg/dL)*	19.0 (7.0-49.0)
Creatinine (mg/dL) *	0.86 (0.44-1.18)
Albumin (g/dL)*	2.6 (1.7-3.8)
Total bilirubin (mg/dL)*	1.6 (0.4-27.1)
Platelet (×10,000/μL) *	8.5 (2.6-37.7)
Urinary specific gravity*	1.016 (1.005-1.050)
Concomitant medication	
Furosemide <40/≥40 mg	32 (38.1%)/52 (61.9%)
Spironolactone <50/≥50 mg	31 (36.9%)/53 (63.1%)

*median (range).

**Table 2:** Differences of characteristics between responders and non-responders

Characteristics	Responders (n=55)	Non-responders (n=29)	*p*-value
Age (years)*	73 (29-93)	71 (35-90)	0.59
Sex (male/female)	36/19	19/10	0.99
BMI (kg/m^2^)*	25.2 (17.4- 35.0)	23.3 (15.5- 36.8)	0.17
Child-Pugh classification (A/B/C)	2/26/27	0/18/11	0.38
Fib-4 index*	8.5 (2.3- 26.0)	8.1 (2.7- 25.8)	0.91
Complication of HCC	27 (49.1%)	18 (62.1%)	0.37
Dose of furosemide (mg/day) *	20.0 (0- 100)	40.0 (0- 100)	0.22
Dose of spironolactone (mg/day)*	25.0 (0- 100)	25.0 (0- 125)	0.84
Na (mEq/L)*	136 (127-143)	136 (120-145)	0.25
K (mEq/L)*	4.0 (3.3-5.2)	4.2 (3.3-5.3)	0.19
Urea nitrogen (mg/dL)*	17.5 (8-43)	23.0 (7-47)	0.03
Creatinine (mg/dL) *	0.84 (0.44-2.52)	0.90 (0.52-1.66)	0.30
Albumin (g/dL)*	2.6 (1.7-3.8)	2.8 (1.8-3.5)	0.76
Total bilirubin (mg/dL)	1.7 (0.4-10.4)	1.5 (0.5-27.1)	0.70
Platelets (x10^4^/μL) *	7.4 (2.2-37.7)	7.7 (3.1-27.6)	0.84
Prothrombin time (%)*	74 (22-117)	67 (35-103)	0.47
Urinary specific gravity*	1.018 (1.005-1.050)	1.013 (1.007-1.024)	0.01

*median (range).

**Table 3:** Univariate and multivariate analysis of factors predicting response to tolvaptan

	Univariate analysis			Multivariate analysis	
	Hazard ratio (95% CI)	*p*-value		Hazard ratio (95% CI)	*p*-value
Age (≥75 years)	1.18 (0.48-2.93)	0.72			
Sex (male)	0.99 (0.39-2.57)	0.99			
BMI (≥24)	3.11 (1.12-8.63)	0.03		5.14 (1.23-22.10)	0.03
Child-Pugh (classification C)	1.58 (0.63-3.95)	0.33			
Complication of HCC	0.59 (0.24-1.48)	0.26			
Serum sodium (≥ 133 mEq/L)	2.24 (0.74-6.77)	0.15		4.40 (0.52-37.20)	0.15
Serum creatinine (≥ 0.8 mg/dL)	0.411 (0.15-1.12)	0.08		1.35 (0.17-10.51)	0.08
Serum urea nitrogen (≥ 21 mg/dL)	0.493 (0.197-1.23)	0.13		0.60 (0.08-4.25)	0.61
Serum albumin (≥ 2.5 g/dL)	0.258 (0.07-0.97)	0.04		0.43 (0.02-8.45)	0.68
Serum total bilirubin (≥ 1.6 mg/dL)	1.83 (0.74-4.55)	0.19		2.44 (0.34-17.00)	0.48
Platelet (≥ 8×10^4^/μL)	1.20 (0.49-2.94)	0.70			
Urinary specific gravity (≥ 1.018)	5.75 (1.60-20.7)	<0.01		6.91 (1.30-36.6)	0.04

**Table 4:** Univariate and multivariate analysis of factors predicting OS

	Univariate analysis		Multivariate analysis	
	Hazard ratio (95% CI)	*p*-value	Hazard ratio (95% CI)	*p*-value
Age (≥70 years)	0.72 (0.39-1.30)	0.27		
Sex (male)	1.21 (0.64-2.29)	0.55		
Child-Pugh (classification C)	2.25 (1.25-4.05)	<0.01	2.20 (1.03-4.70)	0.04
Complication of HCC	2.12 (1.15-3.90)	0.02	2.73 (1.35-5.52)	<0.01
Serum sodium (≥133 mEq/L)	0.29 (0.14-0.611)	<0.01	0.45 (0.20-1.00)	0.05
Serum creatinine (≥0.8 mg/dL)	1.09 (0.59-2.03)	0.79		
Serum urea nitrogen (≥ 21 mg/dL)	0.80 (0.44-1.46)	0.47		
Serum albumin (≥2.5 g/dL)	0.52 (0.26-1.03)	0.06	0.58 (0.26-1.30)	0.19
Serum total bilirubin (≥1.6 mg/dL)	2.69 (1.44-5.01)	<0.01	1.35 (0.62-2.93)	0.45
Platelets (≥8x10^4^/uL)	1.14 (0.63-2.07)	0.66		
Response to tolvaptan	1.02 (0.53-2.00)	0.95		

**Table 5:** Univariate and multivariate analysis of factors predicting OS in tolvaptan responders (n=55)

	Univariate analysis			Multivariate analysis	
	Hazard ratio (95% CI)	*p*-value		Hazard ratio (95% CI)	*p*-value
Age (≥70 years)	0.67 (0.33-1.35)	0.26			
Sex (male)	1.26 (0.61-2.61)	0.54			
Child-Pugh (classification C)	2.05 (1.01-4.18)	0.05		1.17 (0.29-4.71)	0.83
Complication of HCC	1.70 (0.84-3.45)	0.14		6.63 (1.43-30.78)	0.02
Serum sodium (≥133 mEq/L)	0.39 (0.12-1.04)	0.06		0.00 (0.00-Inf)	1.00
Serum creatinine (≥0.8 mg/dL)	1.04 (0.52-2.10)	0.90			
Serum albumin (≥2.5 g/dL)	0.42 (0.19-0.91)	0.03		0.18 (0.03-1.09)	0.06
Serum total bilirubin (≥1.6 mg/dL)	2.59 (1.22-5.50)	0.01		2.36 (0.38-14.78)	0.36
Platelets (≥8x10^4^/uL)	1.39 (0.65-2.97)	0.39			
Early re-exacerbation	2.83 (1.15-6.99)	0.02		6.28 (1.87-21.07)	<0.01
Reduction of conventional diuretics	0.50 (0.24-1.06)	0.07		0.63 (0.25-1.58)	0.32

## Abbreviations

AASLD: American Association for Study of Liver Diseases
ADPKD: autosomal dominant polycystic kidney disease
BCLC: Barcelona Clinic Liver Cancer
BMI: body mass index
BUN: blood urea nitrogen
CART: concentrated ascites reinfusion therapy
EASL: European Association for the Study of the Liver
HBV: hepatitis B virus
HCV: hepatitis C virus
HCC: hepatocellular carcinoma
JSGE: Japanese Society of Gastroenterology
NASH: non-alcoholic steatohepatitis
OS: overall survival
